# Pulmonary Function Difference in Sasang Constitutional Types

**DOI:** 10.1155/2018/9074613

**Published:** 2018-05-02

**Authors:** Jiwon Yoon, Chae Hun Leem, Jong Yeol Kim

**Affiliations:** ^1^KM Fundamental Research Division, Korea Institute of Oriental Medicine, 1672 Yuseong-daero, Yuseong-gu, Daejeon 34054, Republic of Korea; ^2^Department of Physiology, University of Ulsan College of Medicine, 88 Olympic-ro 43-gil, Songpa-gu, Seoul 05505, Republic of Korea

## Abstract

The purpose of this study was to determine the differences in pulmonary function among Sasang constitutional types in young adults. The Sasang Constitutional Analysis Tool (SCAT), pulmonary function tests (PFTs), and cardiopulmonary exercise tests were conducted in 417 participants from 2009 to 2015. Subjects with the Tae-Eum (TE) type had significantly higher inspiratory capacity (IC) and inspiratory reserve volume (IRV) values than those with the So-Yang (SY) and So-Eum (SE) types (*P* < 0.0001). The TE and SY types showed higher forced vital capacity (FVC) and forced expiratory volume in 1 sec (FEV_1_) values than the SE type (*P* < 0.0001). An increase in IRV and a decrease in expiratory reserve volume (ERV) in TE type males remained even after adjusting for covariate factors. These results indicate that young adults with the TE type have weaker lung function than those with the other constitutional types, suggesting its innate physiological pulmonary features.

## 1. Introduction

Sasang Constitutional Medicine (SCM) is a traditional Korean medicine created by Lee Je-ma (1837–1900) at the end of the 19th century [1]. SCM categorizes humans into four types: So-Eum (SE), So-Yang (SY), Tae-Eum (TE), and Tae-Yang (TY). The physical, psychological, physiological, and pathological characteristics of each constitution differ, and treatment methods such as drugs and acupuncture should also differ according to the constitution [2]. Several cross-sectional studies have shown that the prevalence of chronic diseases, such as hypertension [3], diabetes [4], insulin resistance [5], and metabolic syndrome [6], varies depending on the constitution and that the Sasang constitution is an independent risk factor for these diseases. Particularly, it is reported that the risk of chronic illness is higher in subjects with the TE type than in those with other constitutions [7]. The TE type can be vulnerable to various diseases as a result of constitutional characteristics [8]. The TE type is prone to have a higher body mass index (BMI) and prevalence of abdominal obesity than the other types [9]. According to the SCM visceral theory, the TE type is hypothesized to have a hypoactive-lung and hyperactive-liver system, indicating relatively low energy consumption function and relatively developed energy storage function [10]. Studies supporting the hypoactive-lung theory report low mitochondrial metabolism [11], attenuated cellular energy metabolism [12], and reduced adenosine triphosphate- (ATP-) linked oxygen consumption rates [13] of the TE types.

The pulmonary function test (PFT) is widely used in clinical practice to screen for the presence or absence of pulmonary dysfunction [14]. If measured values, such as forced vital capacity (FVC) and forced expiratory volume in 1 sec (FEV_1_), are decreased, it is defined as pulmonary dysfunction [15]. Several studies in multiple populations have shown that pulmonary dysfunction is closely related to cardiovascular disease [16, 17] and is associated with cardiovascular risk factors such as hypertension [18], diabetes [19], and dyslipidemia [20]. Recent studies have shown that metabolic syndrome, consisting of increased blood pressure, insulin resistance, dyslipidemia, and abdominal obesity, also significantly increases the risk of pulmonary dysfunction [21, 22]. However, studies on pulmonary function according to Sasang constitution have not been published, except for those on chronic obstructive pulmonary disease [23], impaired lung function and metabolic syndrome [24], and the comparison of pulmonary function in middle-aged participants [25].

The relationship between Sasang constitution and pulmonary dysfunction has not yet been clarified. There have been few attempts at scientific and comprehensive pathological interpretation. As the risk of metabolic diseases increases, the importance of the TE type's physiological interpretation also increases.

## 2. Methods

### 2.1. Participants

This cross-sectional study was conducted from 2009 to 2015. All of the questionnaire data and clinical data, including Sasang constitutional type (SCT) and PFT, were compiled from the Korea Constitutional Multicenter Bank (KCMB) of the Korea Institute of Oriental Medicine (KIOM). Using this resource, we collected questionnaire data on 417 subjects who were recruited at Asan Medical Center. The present study population comprised a population-based sample selected from healthy subjects recruited through advertisements at Asan Medical Center. Twenty-six subjects who did not complete the PFT were excluded from the analysis. After applying these exclusions, a total of 391 individuals were analyzed. This study was approved by the Institutional Review Board of Asan Medical Center (IRB number: 2009-0566). All participants agreed to join this study, and written informed consent for participation was obtained.

### 2.2. Sasang Constitutional Analysis Tool (SCAT)

The determination of SCT was performed using the SCAT developed by the KIOM [26]. Facial images, body shapes, voice analysis, and questionnaire responses on personality and physiological symptoms were integrated into a multinomial logistic regression analysis. The diagnostic accuracy of SCAT has been reported to be higher than that of QSCC II, which is commonly used for the diagnosis of SCT [27].

### 2.3. PFT and Cardiopulmonary Exercise Test

The PFT was performed using a Vmax 229 Pulmonary Function/Cardiopulmonary Exercise Testing Instrument (SensorMedics, Yorba Linda, CA, USA). FVC and FEV_1_ were measured at rest. FEV_1_/FVC was calculated as FEV_1_ divided by FVC. Total lung capacity and its subdivisions were measured by the nitrogen washout method with the Vmax 229. PFT was performed in accordance with recommended standards [28]. Maximum oxygen consumption (VO_2max_, mL/kg/min), a measure of cardiorespiratory fitness, was recorded using a Vmax ENCORE 29c indirect calorimeter (SensorMedics) during exercise testing on a treadmill following a standard Bruce protocol [29]. The anaerobic threshold (AT) was determined by the V-slope method and was then confirmed. The time duration from the starting point to the time when the participants demanded to stop was defined as the exercise time (sec).

### 2.4. Anthropometric Data and Biochemical Measurements

BMI was calculated as weight divided by height squared. Waist circumference (cm) was measured at the narrowest point between the lower rib and the iliac crest. Blood pressure was measured with a standard mercury sphygmomanometer on the nondominant arm. Blood samples were analyzed at Seoul Clinical Laboratories (Seoul, South Korea) to determine the levels of fasting plasma glucose, insulin, triglycerides (TGs), and high-density lipoprotein (HDL) cholesterol.

### 2.5. Statistical Analysis

All statistical analyses were performed with SPSS Statistics® version 23 (SPSS, IBM Corp, Armonk, NY, USA). One-way analysis of variance (ANOVA) was conducted to evaluate differences in the general characteristics and cardiorespiratory values across SCTs. Bonferroni's post hoc test was performed for multiple comparisons. The PFT and cardiopulmonary fitness data were compared among SCTs using analysis of covariance (ANCOVA) while adjusting for sex, age, height, weight, and presence of diabetes mellitus (factors that are known to affect PFT values). The participants were defined as having diabetes mellitus if a fasting glucose level exceeded ≥126 mg/dL or 2-hour plasma glucose exceeded ≥200 mg/dL. All *P* values < 0.05 were considered statistically significant.

## 3. Results

### 3.1. General Characteristics of Participants

The general characteristics of the participants are shown in Table 1. Overall, height was higher in subjects with the TE type. However, no significant difference was found when stratified by gender. Except for fasting glucose, parameters related to metabolic syndrome, such as weight, BMI, waist circumference, fasting insulin, TGs, and blood pressure, were significantly higher in subjects with the TE type. When stratified by gender, no significant difference was found in either systolic blood pressure (SBP) or diastolic blood pressure (DBP).

### 3.2. PFT Results among SCTs (ANOVA)

Overall, the results of the PFT showed that subjects with the TE type had significantly higher IC and IRV values than those with the SE and SY types. Vital capacity (VC), FVC, and FEV_1_ were lower in subjects with the SE type than in those with the TE and SY types. When stratified by gender, male TE participants had higher inspiratory capacity (IC) and inspiratory reserve volume (IRV) values and lower expiratory reserve volume (ERV) values than those with the other types. Female TE and SY participants had higher VC, inspiratory capacity (IC), and FVC values than those with the SE types.

Interestingly, FVC and FEV_1_ showed a similar trend in the TE and SY groups despite the difference in BMI and TE groups were higher in the SE group. When stratified by gender, IC, ERV, and IRV showed a similar trend despite the difference in BMI; male TE participants had higher IC and IRV values and lower ERV values than the SE type. In females, this tendency was maintained; TE and SY participants had similar VC, IC, and FVC values, which were higher than in the SE participants (Table 2).

### 3.3. PFT Results among SCTs (ANCOVA)

Unlike the ANOVA analysis, in ANCOVA in which the variables (age, height, weight, and presence of diabetes mellitus) that can affect the PFT were adjusted, the influence of the significant variables from the ANOVA almost disappeared.

ANCOVA revealed no significant difference in any PFT variables except for FEV_1_, although there was a trend towards higher IRV and lower ERV and FVC values in subjects with the TE than in other types.

According to the analysis stratified by gender, there was no significant difference in any variable in males. However, although statistical significance was not achieved among the constitutional types, there was a tendency for male TE participants to have lower tidal volume (VT), VC, ERV, and FVC values and higher IC and IRV values than the other constitutional types.

In females, VC and exercise time were significantly lower in SE participants than in SY participants. Additionally, ERV was higher and IRV was lower in TE participants, in contrast to the total and male results (Table 3).

## 4. Discussion

Among the SCTs, the constitution that is profoundly related to pulmonary function is the TE type. Subjects with the TE type have weak pulmonary function and strong liver function compared to the other constitutions [10]. The purpose of this study was to investigate the relationship between pulmonary function and physiological differences among SCTs, especially the TE type.

Gender, age, and height are the major determinants of pulmonary function in normal adults [30, 31]. It has also been reported that lung function deteriorates with high BMI and obesity [32]. In this study, there were significant differences in height, weight, blood pressure, fasting insulin, and HDL values according to the constitution but no differences in age. Therefore, we compared the difference of each constitution before and after adjusting for the above variables as covariates.

When comparing the general characteristics according to constitution, participants with the TE type had a higher body weight, BMI, and waist circumference than the other constitutions; thus, high blood pressure, fasting insulin, TGs, and low HDL were confirmed. This is consistent with previous studies that reported that subjects with the TE type are prone to obesity and are therefore vulnerable to metabolic syndrome, hypertension, and diabetes [6, 9]. The sexually stratified results showed the same tendency. TE type males had significantly higher body weight, BMI, waist circumference, fasting insulin, and TGs and lower HDL values. This tendency was maintained for TE type females, where a higher weight, BMI, blood pressure, waist circumference, fasting insulin, and TGs were distinguished. Considering that these variables distinguished by constitution affect pulmonary function, we took into account parameters such as sex, age, height, weight, and presence of diabetes mellitus as covariates and compared those results with those obtained from one-way ANOVA.

The present study found that, in general, subjects with the TE type exhibit increased pulmonary function and lung volume compared with the other types. Because lung function is mostly affected by height [33], a relatively taller height in TE type subjects may explain the difference in pulmonary capacity. Subjects with the TE type had relatively higher FVC and FEV_1_ values, consistent with previous research that TE type males had a large PFT value [34]. Additionally, the FVC and FEV_1_ values of subjects with the SE type were the smallest among the SCTs. It is presumed that this result is because the height of SE subjects is significantly smaller than those with the TE and SY types.

After adjusting for age, height, weight, and presence of diabetes mellitus as covariates, there was a tendency towards lower FVC values in TE type males than other constitutions, although the tendency was not significant. These results indicate that innate pulmonary function of the TE type is weaker than that of the other constitutions and that the lung function characteristics of the TE type are not the same as those of a general obese group (i.e., subjects with the TE type have unique characteristics in relation to pulmonary function and BMI).

Previous studies have shown that an increase in BMI is associated with a decrease in FVC and FEV_1_ [35]. The TE type subjects have a higher BMI than those from the other groups, with an average of over 25, which is considered obese in Korea [36]. Obesity is closely related to decreased lung function, and it is reported that the distribution of body fat affects pulmonary function in particular [35, 37, 38]. Truncal obesity physically reduces the compliance of the chest wall, respiratory muscular strength and function, and the lung volume and peripheral airway size, and abdominal fat affects the thorax and diaphragm to restrict lung expansion [39]. In addition, the inverse association between waist circumference and pulmonary function in the normal, overweight, and obesity groups suggests a risk that pulmonary function may be reduced by this physical mechanism, which is a tendency that results from the increased waist circumference in TE type subjects [40].

In addition, changes in respiratory function in the TE type reflect altered lung volume caused by obesity. It is reported that obesity causes a reduction in the ERV as a result of a reduction in diaphragm mobility and pulmonary compliance [41]. Obesity also contributes to an increase in IRV to compensate the ventilation [42]. IRV was increased in the total TE group, and a reduction in ERV and an increase in IRV were observed in the male TE group. Multiple studies have demonstrated that the effect of obesity on lung function is greater in males than in females, possibly because of gender-related differences in fat distribution [39]. Even after adjusting for possible covariates, this tendency remained in TE type males (not significant). According to ANOVA, VO_2max_ and exercise time also showed the characteristic of decreased cardiopulmonary function in TE type participants compared to the other types. This result suggests that subjects with the TE type exhibit characteristics of obesity; thus, their cardiorespiratory function is reduced [43].

The current study is an analysis of young adult participants. In the case of middle-aged adults reported previously, a reduction of FVC in the TE types had a stronger tendency than in the present study [25]. Taken together, we believe that the TE type causes slightly weaker pulmonary function in young adults and, as age increases, lung function of subjects with the TE type deteriorates more rapidly than those with the other constitutions.

The following points are limitations to present study. First, we recruited participants who were considered healthy and did not perform any other chest X-rays. Second, variables such as factors affecting pulmonary function (smoking, drinking, and exercise) were not considered together. Thus, a more accurate comparison of pulmonary function was limited. In addition, considering the fact that pulmonary function reaches its maximum value in the mid-20's and declines as age increases [44], it is necessary to have a prospective cohort study and cross-sectional studies.

As mentioned above, subjects with the TE type have the highest prevalence of metabolic syndrome and hypertension than those with the other constitutions [3, 6]. Both metabolic syndrome and pulmonary dysfunction increase the risk of cardiovascular morbidity and mortality [21]. Therefore, for the management of cardiovascular disease, it is necessary to manage and prevent metabolic syndrome with selective PFTs at a relatively young age in people with the TE types.

In conclusion, our data suggest that subjects with the TE type, especially males, exhibit weaker pulmonary function, in terms of lung capacity and volume than those with the SE and SY types.

## Figures and Tables

**Table 1:** General characteristics of participants among the Sasang constitutional types.

Variable	TE	SE	SY	*P* value	Post hoc
Total	153	72	166		
Number of cases					
Age (yr)	31.49 ± 6.84	32 ± 7.12	29.83 ± 6.63	0.0287	
Height (cm)	168.77 ± 8.7	164.48 ± 8.62	167.22 ± 8.6	0.0025	TE > SE
Weight (kg)	73.91 ± 12.08	54.53 ± 8.07	62.62 ± 10.04	<0.0001	TE > SY > SE
BMI (kg/m2)	25.84 ± 2.97	20.06 ± 1.55	22.26 ± 2.02	<0.0001	TE > SY > SE
SBP	109.61 ± 13.09	102.35 ± 11.89	104.52 ± 12.35	<0.0001	TE > SE, SY
DBP	68.46 ± 9.27	63.79 ± 9.2	64.25 ± 8.31	<0.0001	TE > SE, SY
Waist	88.58 ± 8	73.83 ± 5.46	78.33 ± 6.28	<0.0001	TE > SY > SE
Fasting insulin (uU/mL)	9.72 ± 7.19	5.46 ± 3.36	6.39 ± 5.18	<0.0001	TE > SE, SY
TG (mg/dL)	111.24 ± 83.41	70.65 ± 39.15	80.74 ± 43.78	<0.0001	TE > SE, SY
Fasting glucose (mg/dL)	88.23 ± 25.92	85.63 ± 18.53	83.92 ± 16.48	0.1872	
HDL	48.99 ± 17.84	57.87 ± 19.02	55.27 ± 18.46	<0.0001	TE > SE, SY

Male	89	26	89		
Number of cases					
Age (yr)	30.58 ± 6.75	30.54 ± 6.08	29.42 ± 6	0.4355	
Height (cm)	174.59 ± 5.27	173.04 ± 5.71	173.44 ± 5.49	0.2611	
Weight (kg)	81.02 ± 9.45	62.5 ± 6.19	70.4 ± 6.49	<0.0001	TE > SY > SE
BMI (kg/m2)	26.56 ± 2.68	20.83 ± 1.22	23.39 ± 1.74	<0.0001	TE > SY > SE
SBP	113.09 ± 12.2	108.62 ± 11.05	110.93 ± 11.8	0.1932	
DBP	70.17 ± 9.84	67.73 ± 10.3	67.24 ± 8.53	0.1018	
Waist	90.9 ± 7.24	75.91 ± 4.97	81.64 ± 5.21	<0.0001	TE > SY > SE
Fasting insulin (uU/mL)	9.79 ± 6.28	4.57 ± 2.05	6.75 ± 4.85	<0.0001	TE > SE, SY
TG (mg/dL)	115.56 ± 66.97	87.07 ± 52.23	91.64 ± 51.67	0.0116	TE > SY
Fasting glucose (mg/dL)	86.22 ± 22.85	88.02 ± 20.09	85.18 ± 17.15	0.8123	
HDL	44.42 ± 16.74	53.56 ± 17.5	49.89 ± 16.02	0.0174	TE < SY

Female	64	46	77		
Number of cases					
Age (yr)	32.75 ± 6.82	32.83 ± 7.58	30.3 ± 7.3	0.0707	
Height (cm)	160.68 ± 5.43	159.63 ± 5.72	160.02 ± 5.29	0.5867	
Weight (kg)	64.03 ± 7.53	50.02 ± 4.91	53.63 ± 4.14	<0.0001	TE > SY > SE
BMI (kg/m2)	24.84 ± 3.09	19.62 ± 1.55	20.95 ± 1.44	<0.0001	TE > SY > SE
SBP	104.77 ± 12.82	98.8 ± 10.94	97.1 ± 8.11	<0.0001	TE > SE, SY
DBP	66.08 ± 7.89	61.57 ± 7.78	60.81 ± 6.57	<0.0001	TE > SE, SY
Waist	85.35 ± 7.93	72.65 ± 5.41	74.51 ± 5.17	<0.0001	TE > SE, SY
Fasting insulin (uU/mL)	9.63 ± 8.34	5.97 ± 3.84	5.98 ± 5.54	0.0011	TE > SE, SY
TG (mg/dL)	105.24 ± 102.26	61.37 ± 25.68	68.15 ± 27.75	0.0004	TE > SE, SY
Fasting glucose (mg/dL)	91.01 ± 29.64	84.28 ± 17.67	82.46 ± 15.66	0.0616	
HDL	55.34 ± 17.48	60.3 ± 19.59	61.5 ± 19.22	0.1365	

Data are presented as the mean ± standard deviation. *P* value: ANOVA result. TE: Tae-Eum, SE: So-Eum, SY: So-Yang, BMI: body mass index, SBP: systolic blood pressure, DBP: diastolic blood pressure, TG: triglyceride, and HDL: high-density lipoprotein.

**Table 2:** Results of pulmonary function test among Sasang constitutional type (ANOVA).

Variables	TE	SE	SY	*P* value	Post hoc
Total	153	72	166		
Number of subjects					
VT, liters	0.66 ± 0.28	0.58 ± 0.25	0.66 ± 0.30	0.0543	
VC, liters	4.34 ± 0.94	3.79 ± 1.06	4.25 ± 0.93	<0.0001	TE > SE, SY > SE
IC, liters	2.85 ± 0.74	2.27 ± 0.59	2.64 ± 0.66	<0.0001	TE > SY > SE
ERV, liters	1.49 ± 0.52	1.53 ± 0.60	1.61 ± 0.52	0.1574	
IRV, liters	2.20 ± 0.70	1.69 ± 0.53	1.98 ± 0.61	<0.0001	TE > SY > SE
FVC, liters	4.37 ± 0.93	3.85 ± 1.00	4.30 ± 0.95	<0.0001	TE > SE, SY > SE
FEV_1_, liters	3.52 ± 0.75	3.11 ± 0.81	3.51 ± 0.79	<0.0001	TE > SE, SY > SE
FEV_1_/FVC (%)	81.12 ± 8.08	81.06 ± 9.11	82.02 ± 8.46	0.6973	
VO_2max_ (ml/kg/min)	39.82 ± 7.21	40.37 ± 7.39	42.56 ± 7.52	0.0031	SY > TE
AT (mL/(kg·min))	22.56 ± 3.85	23.07 ± 5.32	23.32 ± 4.58	0.3117	
Exercise time (sec)	643.21 ± 86.77	619.88 ± 95.59	672.67 ± 89.82	<0.0001	SY > SE, SY > TE

Male	89	26	89		
Number of subjects					
VT, liters	0.72 ± 0.29	0.71 ± 0.27	0.73 ± 0.32	0.9788	
VC, liters	4.95 ± 0.71	4.86 ± 0.94	4.95 ± 0.60	0.7738	
IC, liters	3.35 ± 0.52	2.85 ± 0.44	3.07 ± 0.52	<0.0001	TE > SY, TE > SE
ERV, liters	1.60 ± 0.59	2.01 ± 0.69	1.87 ± 0.48	0.0005	TE < SY, TE < SE
IRV, liters	2.63 ± 0.53	2.14 ± 0.46	2.34 ± 0.53	<0.0001	TE > SY, TE > SE
FVC, liters	4.96 ± 0.68	4.85 ± 0.82	4.99 ± 0.64	0.1977	
FEV_1_, liters	3.98 ± 0.55	3.86 ± 0.68	4.05 ± 0.57	0.1045	
FEV_1_/FVC (%)	80.78 ± 7.94	79.87 ± 10.24	81.58 ± 8.11	0.7726	
VO_2max_ (ml/kg/min)	43.43 ± 6.45	46.39 ± 6.75	46.34 ± 6.80	0.0086	SY > TE
AT (mL/(kg·min))	23.66 ± 4.03	25.87 ± 6.71	24.83 ± 4.66	0.0676	
Exercise time (sec)	685.06 ± 77.65	689.54 ± 89.78	719.84 ± 90.81	0.0203	SY > TE

Female	64	46	77		
Number of subjects					
VT, liters	0.57 ± 0.24	0.51 ± 0.21	0.58 ± 0.26	0.0844	
VC, liters	3.52 ± 0.49	3.19 ± 0.49	3.44 ± 0.48	0.0002	TE > SE, SY > SE
IC, liters	2.19 ± 0.38	1.94 ± 0.35	2.14 ± 0.39	0.0009	TE > SE, SY > SE
ERV, liters	1.35 ± 0.38	1.25 ± 0.30	1.30 ± 0.39	0.2385	
IRV, liters	1.62 ± 0.45	1.43 ± 0.36	1.56 ± 0.40	0.0681	
FVC, liters	3.54 ± 0.52	3.24 ± 0.47	3.47 ± 0.47	0.0018	TE > SE, SY > SE
FEV_1_, liters	2.88 ± 0.46	2.65 ± 0.48	2.86 ± 0.47	0.0131	TE > SE
FEV_1_/FVC (%)	81.60 ± 8.31	81.79 ± 8.38	82.55 ± 8.89	0.8265	
VO_2max_ (mL/kg/min)	34.81 ± 4.85	36.97 ± 5.30	38.18 ± 5.76	0.0012	SY > TE
AT (mL/(kg·min))	21.03 ± 3.00	21.48 ± 3.54	21.57 ± 3.82	0.6382	
Exercise time (sec)	585.02 ± 61.9	580.5 ± 74.5	618.14 ± 48.46	0.0006	SY > TE, SY > SE

Data are presented as the mean ± standard deviation. *P* value: ANOVA result. TE: Tae-Eum, SE: So-Eum, SY: So-Yang, VT: tidal volume, VC: vital capacity, IC: inspiratory capacity, ERV: expiratory reserve volume, IRV: inspiratory reserve volume, FVC: forced vital capacity, FEV_1_: forced expiratory volume in 1 sec, VO_2max_: maximal oxygen consumption, and AT: anaerobic threshold.

**Table 3:** Results of the pulmonary function test among Sasang constitutional types (ANCOVA).

Variables	TE	SE	SY	*P* value	Post hoc
Total (with sex)	153	72	166		
Number of subjects					
VT, liters	0.63 ± 0.03	0.63 ± 0.04	0.67 ± 0.02	0.4283	
VC, liters	4.18 ± 0.05	4.11 ± 0.07	4.26 ± 0.04	0.1140	
IC, liters	2.67 ± 0.04	2.58 ± 0.06	2.69 ± 0.03	0.2174	
ERV, liters	1.52 ± 0.05	1.53 ± 0.06	1.57 ± 0.04	0.6807	
IRV, liters	2.04 ± 0.04	1.95 ± 0.06	2.02 ± 0.04	0.4930	
FVC, liters	4.18 ± 0.05	4.13 ± 0.07	4.3 ± 0.04	0.0195	
FEV_1_, liters	3.4 ± 0.05	3.31 ± 0.06	3.49 ± 0.04	0.0214	SY > SE
FEV_1_/FVC (%)	81.89 ± 0.84	80.28 ± 1.15	81.39 ± 0.68	0.5863	
VO_2max_ (mL/(kg·min))	41.06 ± 0.57	40.06 ± 0.79	41.56 ± 0.47	0.2009	
AT (mL/(kg·min))	22.89 ± 0.41	23.14 ± 0.57	22.98 ± 0.34	0.9485	
Exercise time (sec)	653.43 ± 7.29	618.87 ± 10.02	663.69 ± 5.95	0.0002	TE > SE, SY > SE

Male	89	26	89		
Number of subjects					
VT, liters	0.69 ± 0.04	0.78 ± 0.07	0.74 ± 0.03	0.4619	
VC, liters	4.88 ± 0.07	4.97 ± 0.13	4.99 ± 0.07	0.5799	
IC, liters	3.22 ± 0.06	3.07 ± 0.11	3.15 ± 0.05	0.5758	
ERV, liters	1.67 ± 0.06	1.9 ± 0.12	1.84 ± 0.06	0.1486	
IRV, liters	2.53 ± 0.06	2.29 ± 0.11	2.4 ± 0.06	0.2204	
FVC, liters	4.87 ± 0.07	4.9 ± 0.12	5.06 ± 0.06	0.0930	
FEV_1_, liters	3.95 ± 0.06	3.87 ± 0.12	4.09 ± 0.06	0.0892	
FEV_1_/FVC (%)	81.51 ± 1.03	78.96 ± 1.86	80.93 ± 0.91	0.5394	
VO_2max_ (mL/(kg·min))	45.01 ± 0.79	44.2 ± 1.42	45.41 ± 0.69	0.6851	
AT (mL/(kg·min))	24.2 ± 0.59	25.18 ± 1.06	24.49 ± 0.52	0.7638	
Exercise time (sec)	701.51 ± 10.57	664.48 ± 19.1	710.7 ± 9.34	0.0636	

Female	64	46	77		
Number of subjects					
VT, liters	0.58 ± 0.04	0.49 ± 0.04	0.58 ± 0.03	0.1555	
VC, liters	3.42 ± 0.07	3.23 ± 0.07	3.46 ± 0.05	0.0271	SY > SE
IC, liters	2.06 ± 0.06	2.02 ± 0.06	2.18 ± 0.04	0.0373	
ERV, liters	1.39 ± 0.06	1.2 ± 0.06	1.28 ± 0.04	0.1251	
IRV, liters	1.49 ± 0.06	1.53 ± 0.07	1.6 ± 0.05	0.3310	
FVC, liters	3.45 ± 0.07	3.29 ± 0.07	3.47 ± 0.05	0.0823	
FEV_1_, liters	2.82 ± 0.07	2.68 ± 0.07	2.84 ± 0.05	0.1580	
FEV_1_/FVC (%)	82.05 ± 1.43	81.5 ± 1.5	81.96 ± 1.05	0.9614	
VO_2max_ (mL/(kg·min))	37.04 ± 0.84	35.28 ± 0.89	37.35 ± 0.62	0.1232	
AT (mL/(kg·min))	21.45 ± 0.57	21.22 ± 0.6	21.38 ± 0.42	0.9663	
Exercise time (sec)	601.64 ± 9.79	566.46 ± 10.31	612.72 ± 7.17	0.0005	SY > SE

Data are presented as the mean ± standard error. *P* value: ANCOVA test result. Data are adjusted for sex, age, height, weight, and presence of diabetes mellitus. TE: Tae-Eum, SE: So-Eum, SY: So-Yang, VT: tidal volume, VC: vital capacity, IC: inspiratory capacity, ERV: expiratory reserve volume, IRV: inspiratory reserve volume, FVC: forced vital capacity, FEV_1_: forced expiratory volume in 1 sec, VO_2max_: maximal oxygen consumption, and AT: anaerobic threshold.

## Data Availability

Data are available from the Korea Institute of Oriental Medicine (KIOM) Korean Medicine Data Center (KDC, http://kdc.kiom.re.kr/html/, permission number: 20160302) for researchers who meet the criteria for access to confidential data
